# Worldwide transmission of ST11-KL64 carbapenem-resistant *Klebsiella pneumoniae*: an analysis of publicly available genomes

**DOI:** 10.1128/msphere.00173-23

**Published:** 2023-05-18

**Authors:** Junna Wang, Yu Feng, Zhiyong Zong

**Affiliations:** 1 Center of Infectious Diseases, West China Hospital, Sichuan University, Chengdu, China; 2 Center for Pathogen Research, West China Hospital, Sichuan University, Chengdu, China; 3 Division of Infectious Diseases, State Key Laboratory of Biotherapy, Chengdu, China; University of Michigan-Ann Arbor, Ann Arbor, Michigan, USA

**Keywords:** *Klebsiella pneumoniae*, carbapenem resistance, transmission clusters

## Abstract

ST11-KL64 is an internationally distributed lineage of carbapenem-resistant *Klebsiella pneumoniae* and is the most common type in China. The international and interprovincial (in China) transmission of ST11-KL64 CRKP remains to be elucidated. We used both static clusters defined based on a fixed cutoff of ≤21 pairwise single-nucleotide polymorphisms and dynamic groups defined by modeling the likelihood to be linked by a transmission threshold to investigate the transmission of ST11-KL64 strains based on genome sequences mining. We analyzed all publicly available genomes (*n* = 730) of ST11-KL64 strains, almost all of which had known carbapenemase genes with KPC-2 being dominant. We identified 4 clusters of international transmission and 14 clusters of interprovincial transmission across China of ST11-KL64 strains. We found that dynamic grouping could provide further resolution for determining clonal relatedness in addition to the widely adopted static clustering and therefore increases the confidence for inferring transmission.

Carbapenem-resistant *Klebsiella pneumoniae* (CRKP) is a serious challenge for clinical management and is prone to spread in and between healthcare settings. ST11-KL64 is the dominant CRKP type in China with a worldwide distribution. Here, we used two different methods, the widely used clustering based on a fixed single-nucleotide polymorphism (SNP) cutoff and the recently developed grouping by modeling transmission likelihood, to mine all 730 publicly available ST11-KL64 genomes. We identified international transmission of several strains and interprovincial transmission in China of a few, which warrants further investigations to uncover the mechanisms for their spread. We found that static clustering based on ≤21 fixed SNPs is sensitive to detect transmission and dynamic grouping has higher resolutions to provide complementary information. We suggest the use of the two methods in combination for analyzing transmission of bacterial strains. Our findings highlight the need of coordinated actions at both international and interprovincial levels for tackling multi-drug resistant organisms.

## OBSERVATION

Carbapenem-resistant *Klebsiella pneumoniae* (CRKP) is a severe threat for human health globally (1). Currently, available data indicate that the international or regional spread of CRKP is largely driven by certain sequence types (STs) such as ST11, ST15, ST37, ST258, and ST307 (2 - 4). Among these types, ST11 CRKP is mainly seen in Asia and South America (3, 5) and could be assigned to several capsular (KL) types. In particular, ST11-KL64 is the dominant CRKP type at present in China (6) and also appears to be commonly seen in Brazil (7). However, the transmission of ST11-KL64 CRKP in the world and across provinces in China remains to be elucidated. Recently, the national transmission of CRKP in the USA has been addressed using both static clusters defined based on a fixed cutoff of ≤21 pairwise single-nucleotide polymorphisms (SNPs) and dynamic clusters defined by modeling the likelihood to be linked by a transmission threshold (8). We therefore employ the described methodology to investigate the transmission of ST11-KL64 CRKP including the international transmission and the interprovincial transmission in China.

### Thirty-two static clusters and 59 dynamic groups were identified from 730 ST11-KL64 genomes

**Fig 1:**
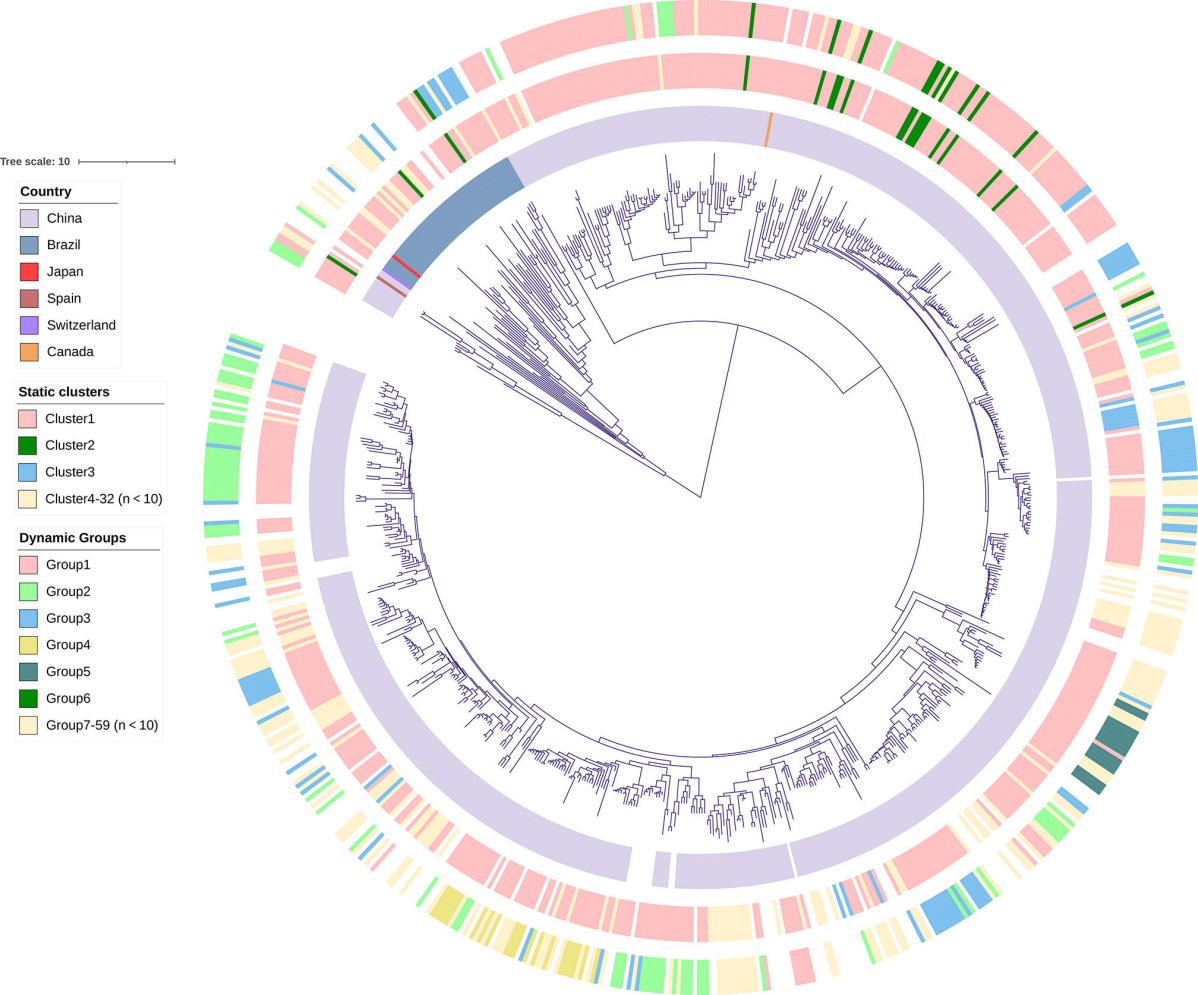
Phylogenomic tree of ST11-KL64 *Klebsiella pneumoniae* strains (*n* = 730). The tree was visualized and annotated using iTOL v3 (15). The circles from the inner to the outer represent country, static clusters based on ≤21 SNPs, dynamic groups based on a *T* = 5 transmission threshold, respectively. Scale bar represents number of nucleotide substitutions per site. Clusters and groups containing ≥ 10 genomes are shown in different colors. Countries where the strain was recovered are indicated. A more detailed figure containing information about the acquired carbapenemases as well as year and source of isolation is shown in Fig. S1. SNP, single-nucleotide polymorphism.

As of 1 June 2022, there were 13,625 *K*. *pneumoniae* genome assemblies in NCBI. By quality control, 1,039 genomes were excluded due to duplicated biosample (*n* = 103), low-quality assembly as defined by NCBI (e.g., excessive frameshifted proteins and fragmented assembly; *n* = 674), < 95% completeness (*n* = 51), > 5% contamination (*n* = 25), > 50% heterogeneity (*n* = 162), or genomes belonging to species other than *K. pneumoniae* (*n* = 24). The remaining 12,586 genomes were included in further study, comprising 730 ST11-KL64 ones with STs and capsular types being determined using Kleborate v2.2.0 (9). Almost all (*n* = 704, 96.4%) of the 730 ST11-KL64 strains had genes encoding known carbapenemases, among which KPC-2 was the most common (94.0%, 686/730). Information about the geographical location was available for 713 out of the 730 genomes with most (*n* = 659; 659/713, 92.4%) seen in China, 50 (7.0%) in Brazil and 1 in each of Canada, Japan, Spain, and Switzerland (Dataset S1). These 730 genome assemblies were subjected to removing recombination regions using Gubbins v3.1.6 (10) and thereafter to calling core SNPs with Snippy (https://github.com/tseemann/snippy) using strain 090357 (accession no. CP066523) as the reference. The phylogeny was built from core SNPs under GTR model with site rate variation and a 100-bootstrap test. A maximum likelihood phylogenomic tree (Fig. 1 for simplified view and Fig. S1 for more details) was therefore inferred with RAxML v8.2.4 (11) using 100 bootstrapping under the GTR model. Static clusters were conducted by R program TransCluster based on a fixed cutoff of ≤21 pairwise SNPs (12). We used the term of dynamic groups instead of dynamic clusters to avoid confusion with static clusters. Dynamic groups were assigned based on modeling the likelihood that isolates were linked by a transmission threshold (*T*), which was calculated by R program TransCluster (12) with combining the rate of SNP accumulation (λ), the collection time of ST11-KL64 strains and an estimated transmission rate (β). A λ value of 10.1 substitutions/genome/year was calculated as previously using paired longitudinal samples of KPC-producing CRKP (13). A β value of 5.8 was estimated previously from epidemiologic investigation of nosocomial *K. pneumoniae* (14). *T* value of 5 was selected according to the previous study of the CRKP transmission in U.S. hospitals (8).

Overall, 32 static clusters and 59 dynamic groups were identified and incorporated 650 (89.0%) and 539 (73.8%) strains, respectively. Among the 32 static clusters, cluster 1 was the largest comprising 515 strains (70.5%) and could be further assigned to 32 dynamic groups (Table S1, listing all static clusters and dynamic groups). Strains of nine static clusters could not be assigned to a dynamic group. The remaining 22 static clusters matched one or two dynamic groups (Table S1). On the other hand, each individual group of the 59 dynamic ones matched a single static cluster only (Table S1). Therefore, dynamic groups appeared to generate complementary, yet overlapping, information for static clusters and provide further resolution to infer transmission of CRKP strains.

We evaluated the impact of applying different SNP cutoffs up to 25 on the number of static clusters and the matching with dynamic groups. When 21 SNPs were applied, 89.0% of all strains could be assigned to a static cluster (Table S2) and all but one dynamic group could match a single cluster (Fig. S2). In contrast, when 15 or 16 SNPs were used to define static clusters, all dynamic groups could match a single static cluster (Fig. S2) but the proportion of strains able to be assigned to a cluster dropped to 84.2% or 85.2% (Table S2). We also evaluated the impact of adjusting the transmission threshold (*T*) and β parameter on dynamic grouping as described previously (7). When a *T* threshold was selected from a wide range (1, 20) with a fixed β value of 5.8 (14) , the number of international groups was 2 (*T* = 20) to 6 (*T* = 4, 5, or 6) and that of interprovince groups was 6 (*T* = 13) to 24 (*T* = 3) (Fig. S3). Conversely, when a β parameter was selected from a wide range (1, 20) with a fixed *T* value of 5 (8), the number of international groups was 2 (β = 1) to 7 (β = 7) and that of interprovince groups was 7 (β = 1 or 3) to 28 (β = 9) (Fig. S3). The above analysis highlights that the choose of parameters (the SNP cutoff, the *T* threshold, and the β parameter) will generate varied numbers of clusters and groups. Nevertheless, although the number of groups and clusters varied with the use of different parameters, both international and interprovince transmission were identified. We therefore performed further analyses based on static clusters defined with a fixed cutoff of ≤21 pairwise SNPs, which was applied previously (12), and dynamic groups generated with a λ value of 10.1 substitutions/genome/year, a β value of 5.8, and a *T* value of 5 as described previously (8).

### Four clusters associated with international transmission

Next, we aligned the geographic information with the clusters and groups to identify transmission. Four static clusters, clusters 1, 2, 5, and 23, comprised strains from more than one country (Table 1), suggesting possible international transmissions. In particular, strains of cluster 1 (93.8% had KPC-2) were from Brazil, Canada, China, Japan, Spain, and Switzerland of four continents. Within cluster 1, there were four dynamic groups (groups 1, 2, 3, and 10) containing strains from China and one additional country, either Brazil (three groups) or Switzerland (one group) with 91.4% to 100% encoding KPC-2. This provides further evidence of international transmissions of certain CRKP strains and suggests China as the possible hotspot of ST11-KL64 strains. Cluster 2 contained 18 strains (16 encoding KPC-2) from China and Brazil with 12 from the two countries belonging to a single dynamic group, supporting the presence of international transmission. Cluster 5 contained four strains from Brazil (three encoding KPC-2 and one OXA-48) and two from China (both encoding KPC-2). Cluster 23 contained one strain from Brazil and one from China, both of which belonged to a common dynamic group and had KPC-2. Notably, strains of cluster 1 from Canada, Japan, or Spain along with some strains from Brazil or China and all six strains of cluster 5 could not be assigned to dynamic groups (Table 1). This illustrates that the use of dynamic groups alone may miss possible international transmission of some strains.

**TABLE 1:** Static clusters and dynamic groups of ST11-KL64 CRKP associated with international and/or interprovincial (in China) transmission^*a*^

Static cluster^*b*^	Dynamic group^*b*^ ^,^ ^*c*^	Country	Provinces of China (no. of genomes)
**1** (515 [17])	**1** (163 [7])	**China, Brazil**	Anhui (4), Beijing (1), Jiangsu (6), Jiangxi (2), Henan (2), Shandong (2), Shanghai (9), Sichuan (80), Taiwan (3), Zhejiang (28)
	**2** (87 [3])	**China, Switzerland**	Beijing (3), Henan (1), Jiangsu (1), Jiangxi (5), Shanghai (4), Shandong (1), Sichuan (12), Taiwan (2), Zhejiang (38)
	**3** (84 [2])	**China, Brazil**	Anhui (2), Jiangsu (2), Guangdong (1), Hunan (1), Shandong (1), Shanghai (2), Sichuan (30), Zhejiang (20)
	5 (16 [2])	China	Anhui (6), Beijing (4), Shandong (2), Shanghai (1), Zhejiang (1)
	8 (8)	China	Shanghai (2), Sichuan (6)
	**10** (6)	**China, Brazil**	Sichuan (4), Zhejiang (1)
	16 (4)	China	Anhui (1), Henan (3)
	27 (3)	China	Sichuan (2), Zhejiang (1)
	47 (2)	China	Shanghai (1), Zhejiang (1)
	– (63 [3])	Brazil, Canada, China, Japan, Spain	Anhui (1), Hunan (1), Jiangsu (2), Shandong (1), Shanghai (1), Sichuan (13), Zhejiang (15)
**2** (18)	**6** (12)	**China, Brazil**	Sichuan (8), Zhejiang (1)
	12 (5)	China	Sichuan (1), Taiwan (1)
	– (1)	Brazil	
3 (14)	9 (7)	China	Sichuan (7)
	43 (2)	China	Zhejiang (2)
	– (5)	China	Sichuan (1), Zhejiang (2)
4 (9 [2])	11 (6 [1])	China	Anhui (1), Henan (1), Shanghai (3), Zhejiang (1)
	57 (2 [1])	China	Henan (1), Jiangsu (1)
	– (1)	China	
**5** (6)	–	**China, Brazil**	Shanghai (1), Zhejiang (1)
7 (6)	15 (4)	China	Sichuan (1), Zhejiang (3)
	– (2)	China	Zhejiang (1)
8 (6)	22 (3)	China	Henan (3)
	46 (2)	China	Zhejiang (2)
	– (1)	China	
9 (4)	– (3)	China	Anhui (1), Zhejiang (2)
10 (4)	– (4)	China	Shanghai (1), Zhejiang (3)
16 (4)	28 (3)	China	Hunan (2), Zhejiang (1)
	– (1)	China	Zhejiang (1)
18 (3)	50 (2)	China	Zhejiang (2)
	– (1)	China	Shandong (1)
20 (3)	– (3)	China	Hunan (1), Zhejiang (1)
21 (3)	– (3)	China	Henan (1), Jiangsu (1), Sichuan (1)
**23** (2)	**33** (**2**)	**China, Brazil**	Zhejiang (1)
25 (2)	– (2)	China	Jiangsu (1), Tianjin (1)

^*a*^ The complete list containing all static clusters and dynamic groups is shown in Table S1. In Table 1, only static clusters containing strains from more than one country or more than one province of China are shown. Within cluster 1, only dynamic groups containing strains from more than one country or more than one province of China are shown. For some strains, the information about the country or the Chinese province is not available. Static clusters and dynamic groups with international distribution are in bold.

^*b*^ (Number of genomes [number of genomes without known carbapenemase-encoding genes; otherwise, all strains had known carbapenemase-encoding genes]).

^*c*^ –, could not be assigned to a dynamic group.

### Fourteen static clusters associated with interprovincial transmission in China

Genome sequences of ST11-KL64 *K. pneumoniae* strains from 16 out of the 34 provincial regions of China were available in GenBank (Dataset S1). Fourteen static clusters comprised strains from two or more provinces of China, suggesting possible interprovincial transmission. The three static clusters and the five dynamic groups associated with international transmission were also found to be associated with interprovincial transmission in China (Table 1). Cluster 1 contained strains from 12 provinces and among the 32 dynamic groups within cluster 1, nine contained strains from more than one province. In particular, three dynamic groups (groups 1, 2, and 3) within cluster 1 contained 84 to 163 strains from 8 to 10 provinces, suggesting wide dissemination across China. Notably, these three dynamic groups were also associated with international transmission and may therefore represent high-risk clones, warranting further studies. Four clusters (2, 4, 7, and 16) contained one or two dynamic groups with strains from more than one province. The remaining nine static clusters comprising strains from two or more provinces contained dynamic groups with strains from a single province or could not be assigned to dynamic groups (Table 1). The possible interprovincial transmission of the nine clusters would be therefore missed by using dynamic clustering alone. This highlights the need of static clusters and dynamic groups to be used in combination for analyzing CRKP transmission.

### Limitations and conclusion

We are aware of the limitations of this analysis. First, publicly available genomes were highly biased and therefore the international and interprovincial transmission here are likely to represent only a tip of an iceberg with wider transmission of more ST11-KL64 strains are waiting for discovery. Second, the geographical locations of the available ST11-KL64 strains largely restricted to China and, to a lesser extent, Brazil. This may imply that ST11-KL64 strains are not yet widely distributed in the world at present but could also result from under sampling. Third, metadata sets of the ST11-KL64 strains are not available, preventing further mining to identify the sources, reconstruct the transmission events, and uncover the drivers for the international and interprovincial transmission. Such mining would provide much-needed information for designing countermeasures. Fourth, as shown above, the number of clusters and groups varies according to the SNP cutoff and the parameters (λ, β, and *T*). In particular, changes of any of the three parameters (λ, β, and *T*) could have an impact on grouping and there are enormous, if not numerous, combinations of different values of the parameters. The ideal SNP cutoff and parameter values remain to be determined. Despite the limitations, the publicly available genomes are still precious sources for analysis to identify transmission of multi-drug resistant organisms and both international and interprovincial transmission of bacterial strains have been identified even when different SNP cutoffs and parameter values were used.

In conclusion, we analyzed 730 publicly available genomes of ST11-KL64 strains, almost all of which had known carbapenemase genes, by two methodologies, static clustering based on a fixed SNP cutoff and dynamic grouping by modeling transmission likelihood. We highlighted that ST11-KL64 is an internationally distributed lineage warranting rigorous monitoring and further studies. We identified international transmission of several ST11-KL64 strains and interprovincial transmission in China of a few strains. We also found several ST11-KL64 strains with particularly wide distribution, which warrants further investigations to uncover the mechanisms for their spread. We argued that dynamic grouping could provide complementary information to enhance the resolution for determining clonal relatedness to the widely adopted method based on a fixed SNP cutoff and the two methods may be used in combination for analyzing transmission of bacterial strains. We believe that coordinated actions at both international and interprovincial levels should be taken to tackle multi-drug resistant organisms like ST11-KL64 CRKP.

## SUPPLEMENTAL MATERIAL

FIG S1 msphere.00173-23-s0001.png Phylogenomic tree of ST11-KL64 *Klebsiella pneumoniae* strains with detailed information. This figure is Fig. 1 in the main text but containing additional data about the acquired carbapenemase type (Carb_acquired), date (year), and isolation source. Scale bar represents number of nucleotide substitutions per site. NA, not available.

FIG S2 msphere.00173-23-s0002.png The number of dynamic groups matching a static cluster when different single-nucleotide polymorphism cutoff was applied for defining the cluster. One-to-one match means that an individual static cluster contains a single dynamic group. Several-to-one match means that an individual static cluster contains two or more dynamic groups.

FIG S3 msphere.00173-23-s0003.png Effects of parameters tuning on dynamic groups. The dynamic groups algorithm was tuned by adjusting either the transmission threshold (T) (panels A and B) or the intermediate transmission rate (β) (panels C and D) and the other parameters (molecular clock rate = 10.1, *T* = 5, or β = 5.8) was constant when applicable. The impact on dynamic groups is summarized as either the distribution of isolates within groups (panels A and C) or the distribution of groups (panels B and D).

DATASET S1 msphere.00173-23-s0004.xls The 730 ST11-KL64 *Klebsiella pneumoniae* genomes and the assignation to clusters and groups.

TABLE S1 msphere.00173-23-s0005.docx The complete list of all static clusters and dynamic groups of ST11-KL64 strains.

TABLE S2 msphere.00173-23-s0006.docx The number of static clusters based on different single-nucleotide polymorphism cutoffs and the comparison between static clusters and dynamic groups.

ASM does not own the copyrights to Supplemental Material that may be linked to, or accessed through, an article. The authors have granted ASM a non-exclusive, world-wide license to publish the Supplemental Material files. Please contact the corresponding author directly for reuse.
