# Supplementary figures and images for: Worldwide transmission of ST11-KL64 carbapenem-resistant Klebsiella pneumoniae: an analysis of publicly available genomes
Source: mSphere. 2023 May 18;8(4):e00173-23. doi: 10.1128/msphere.00173-23 (PMC10449508; doi:10.1128/msphere.00173-23)

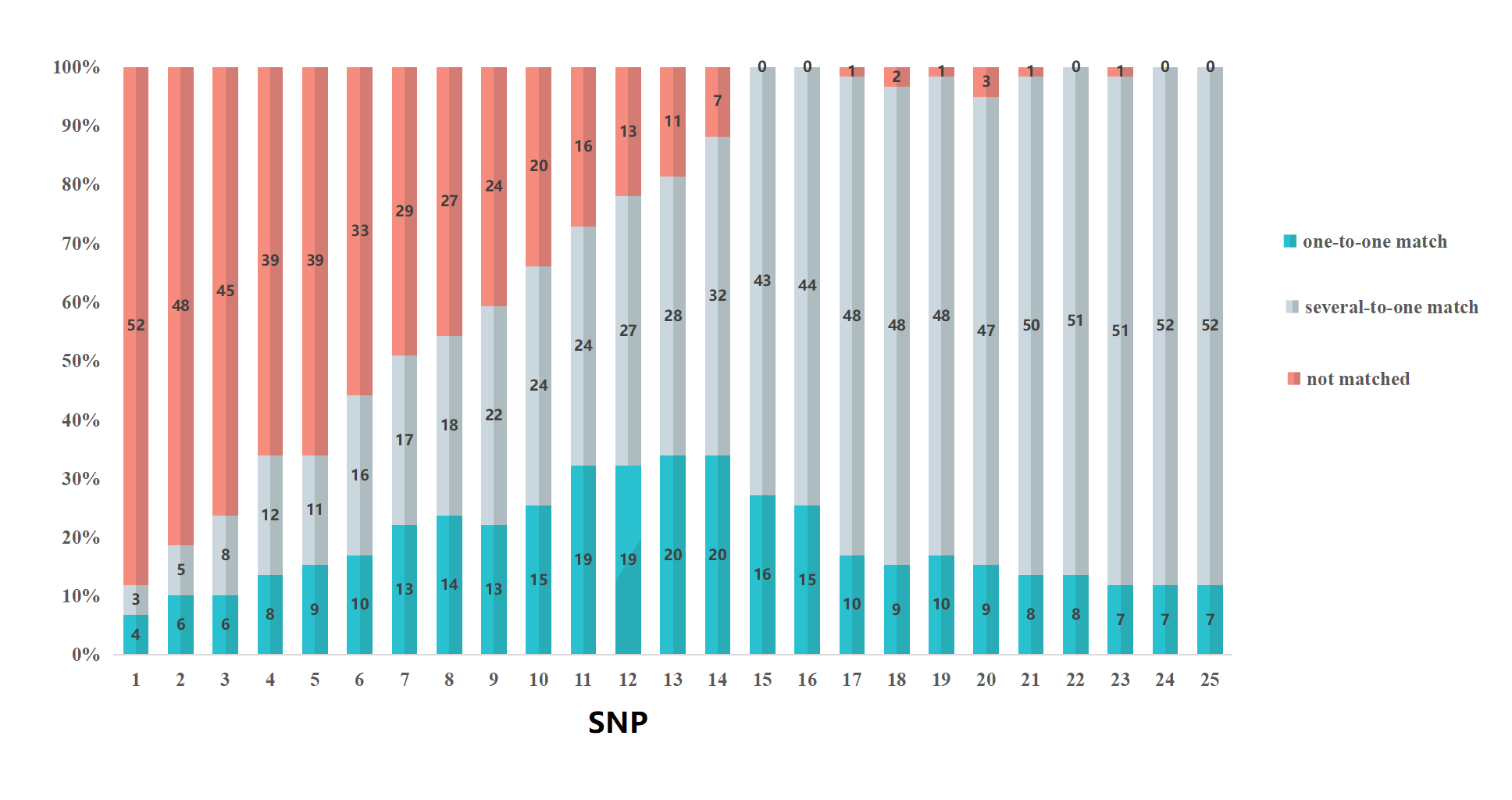

Supplement: FIG S2 — The number of dynamic groups matching a static cluster when different single-nucleotide polymorphism cutoff was applied for defining the cluster. One-to-one match means that an individual static cluster contains a single dynamic group. Several-to-one match means that an individual static cluster contains two or more dynamic groups. [file msphere.00173-23-s0002.png]

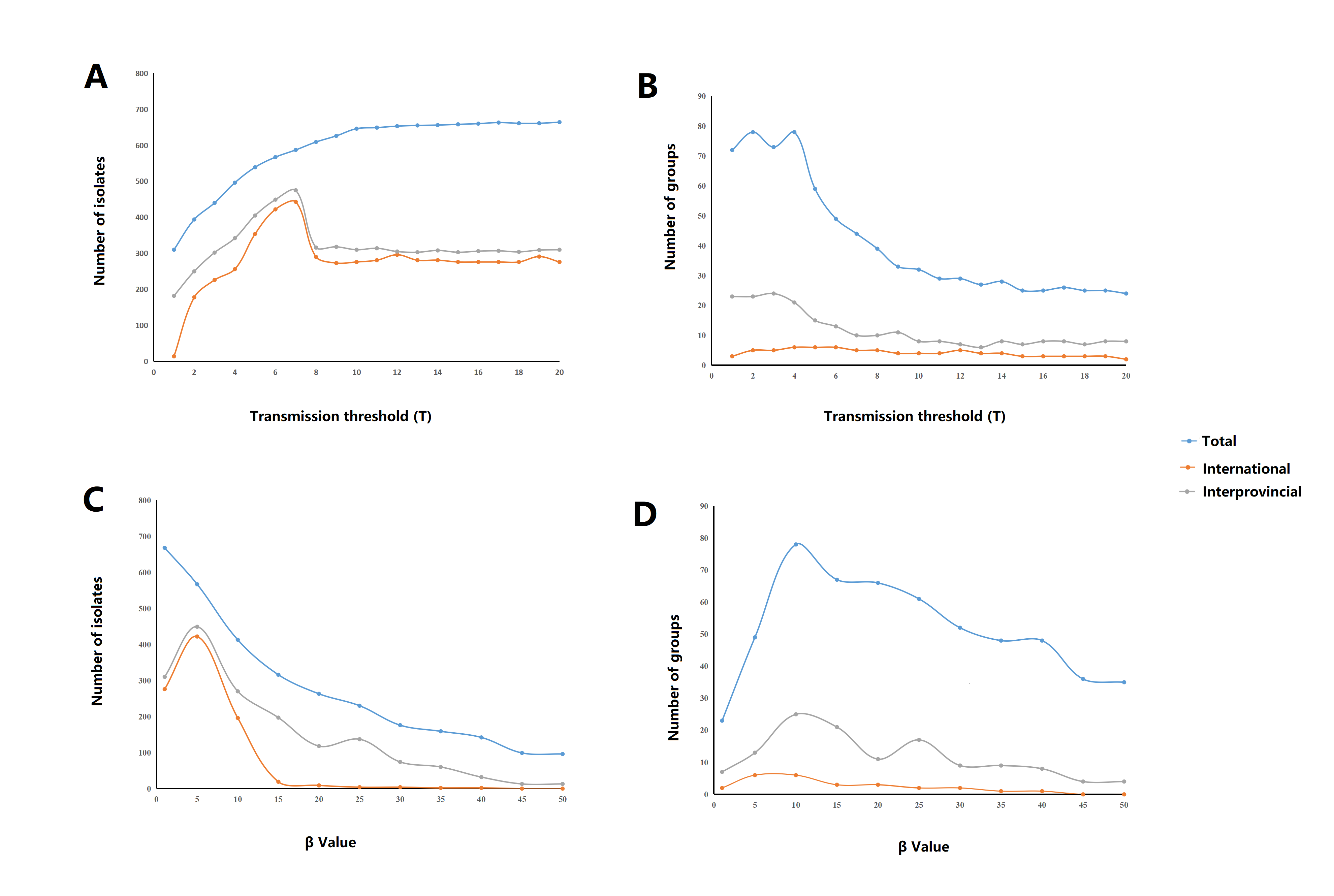

Supplement: FIG S3 — Effects of parameters tuning on dynamic groups. The dynamic groups algorithm was tuned by adjusting either the transmission threshold (T) (panels A and B) or the intermediate transmission rate (β) (panels C and D) and the other parameters (molecular clock rate = 10.1, T = 5, or β = 5.8) was constant when applicable. The impact on dynamic groups is summarized as either the distribution of isolates within groups (panels A and C) or the distribution of groups (panels B and D). [file msphere.00173-23-s0003.png]
