# Supplementary material for: Worldwide transmission of ST11-KL64 carbapenem-resistant Klebsiella pneumoniae: an analysis of publicly available genomes
Source: mSphere. 2023 May 18;8(4):e00173-23. doi: 10.1128/msphere.00173-23 (PMC10449508; doi:10.1128/msphere.00173-23)
Supplement: TABLE S1 — The complete list of all static clusters and dynamic groups of ST11-KL64 strains. [file msphere.00173-23-s0005.docx]

Table S1. The complete list of all static clusters and dynamic groups of ST11-KL64 strains.

| Static cluster | No., strains | Dynamic cluster | No., strains | Country | Provinces of China (no. of genomes) |
| --- | --- | --- | --- | --- | --- |
| 1 | 515 | 1 | 163 | China, Brazil | Anhui (4), Beijing (1), Henan (2), Jiangsu (6), Jiangxi (2), Shandong (2), Shanghai (9), Sichuan (80), Taiwan (3), Zhejiang (28) |
|  |  | 2 | 87 | China, Switzerland | Beijing (3), Henan (1), Jiangsu (1), Jiangxi (5), Shanghai (4), Shandong (1), Sichuan (12), Taiwan (2), Zhejiang (38) |
|  |  | 3 | 84 | China, Brazil | Anhui (2), Jiangsu (2), Guangdong (1), Hunan (1), Shandong (1), Shanghai (2), Sichuan (30), Zhejiang (20) |
|  |  | 4 | 20 | China | Zhejiang (12) |
|  |  | 5 | 16 | China | Anhui (6), Beijing (4), Shandong (2), Shanghai (1), Zhejiang (1) |
|  |  | 7 | 8 | China | Hunan (6) |
|  |  | 8 | 8 | China | Shanghai (2), Sichuan (6) |
|  |  | 10 | 6 | China, Brazil | Sichuan (4), Zhejiang (1) |
|  |  | 13 | 5 | China | Sichuan (2) |
|  |  | 16 | 4 | China | Anhui (1), Henan (3) |
|  |  | 17 | 4 | China | Zhejiang (2) |
|  |  | 20 | 3 | China | - |
|  |  | 23 | 3 | China | Zhejiang (2) |
|  |  | 25 | 3 | China | Taiwan (1) |
|  |  | 26 | 3 | China | Sichuan (3) |
|  |  | 27 | 3 | China | Sichuan (2), Zhejiang (1) |
|  |  | 29 | 2 | Brazil | - |
|  |  | 30 | 2 | Brazil | - |
|  |  | 32 | 2 | Brazil | - |
|  |  | 34 | 2 | China | Sichuan (1) |
|  |  | 36 | 2 | China | Sichuan (2) |
|  |  | 40 | 2 | China | Zhejiang (1) |
|  |  | 42 | 2 | China | Zhejiang (1) |
|  |  | 44 | 2 | China | Zhejiang (1) |
|  |  | 45 | 2 | China | Zhejiang (2) |
|  |  | 47 | 2 | China | Shanghai (1), Zhejiang (1) |
|  |  | 48 | 2 | China | Zhejiang (1) |
|  |  | 51 | 2 | China | Zhejiang (1) |
|  |  | 52 | 2 | China | Zhejiang (2) |
|  |  | 54 | 2 | China | - |
|  |  | 56 | 2 | China | - |
|  |  | 59 | 2 | China | Shanghai (2) |
|  |  | - | 63 | Brazil, Canada, China, Japan, Spain | Anhui (1), Hunan (1), Jiangsu (2), Shandong (1), Shanghai (1), Sichuan (13), Zhejiang (15) |
| 2 | 18 | 6 | 12 | China, Brazil | Sichuan (8), Zhejiang (1) |
|  |  | 12 | 5 | China | Sichuan (1), Taiwan (1) |
|  |  | - | 1 | Brazil |  |
| 3 | 14 | 9 | 7 | China | Sichuan (7) |
|  |  | 43 | 2 | China | Zhejiang (2) |
|  |  | - | 5 | China | Sichuan (1), Zhejiang (2) |
| 4 | 9 | 11 | 6 | China | Anhui (1), Henan (1), Shanghai (3), Zhejiang (1) |
|  |  | 57 | 2 | China | Henan (1), Jiangsu (1) |
|  |  | - | 1 | China |  |
| 5 | 6 | - | - | China, Brazil | Shanghai (1), Zhejiang (1) |
| 6 | 6 | 18 | 4 | China | - |
|  |  | - | 2 | China | Sichuan (1) |
| 7 | 6 | 15 | 4 | China | Sichuan (1), Zhejiang (3) |
|  |  | - | 2 | China | Zhejiang (1) |
| 8 | 6 | 22 | 3 | China | Henan (3) |
|  |  | 46 | 2 | China | Zhejiang (2) |
|  |  | - | 1 | China |  |
| 9 | 4 | - | - | China | Anhui (1), Zhejiang (2) |
| 10 | 4 | - | - | China | Shanghai (1), Zhejiang (3) |
| 11 | 4 | 14 | 4 | China | Sichuan (4) |
| 12 | 4 | 38 | 2 | China | Sichuan (2) |
|  |  | 39 | 2 | China | Sichuan (2) |
| 13 | 4 | 21 | 3 | China | - |
|  |  | - | 1 | China | - |
| 14 | 4 | 24 | 3 | China | Zhejiang (2) |
|  |  | - | 1 | China | Zhejiang (1) |
| 15 | 4 | 49 | 2 | China | Zhejiang (2) |
|  |  | - | 2 | China | Zhejiang (1) |
| 16 | 4 | 28 | 3 | China | Hunan (2), Zhejiang (1) |
|  |  | - | 1 | China | Zhejiang (1) |
| 17 | 3 | 19 | 3 | China | Sichuan (3) |
| 18 | 3 | 50 | 2 | China | Zhejiang (2) |
|  |  | - | 1 | China | Shandong (1) |
| 19 | 3 | 31 | 2 | Brazil | - |
|  |  | - | 1 | Brazil | - |
| 20 | 3 | - | - | China | Hunan (1), Zhejiang (1) |
| 21 | 3 | - | - | China | Henan (1), Jiangsu (1), Sichuan (1) |
| 22 | 3 | 58 | 2 | China | Zhejiang (1) |
|  |  | - | 1 | China | Zhejiang (1) |
| 23 | 2 | 33 | 2 | China, Brazil | Zhejiang (1) |
| 24 | 2 | - | - | Brazil | - |
| 25 | 2 | - | - | China | Jiangsu (1), Tianjin (1) |
| 26 | 2 | 35 | 2 | China | Sichuan (2) |
| 27 | 2 | 37 | 2 | China | Sichuan (2) |
| 28 | 2 | - | - | China | Anhui (1) |
| 29 | 2 | 53 | 2 | China | - |
| 30 | 2 | 55 | 2 | China | Zhejiang (1) |
| 31 | 2 | 41 | 2 | China | Shanghai (2) |
| 32 | 2 | - | - | China | - |
