# Supplementary material for: Worldwide transmission of ST11-KL64 carbapenem-resistant Klebsiella pneumoniae: an analysis of publicly available genomes
Source: mSphere. 2023 May 18;8(4):e00173-23. doi: 10.1128/msphere.00173-23 (PMC10449508; doi:10.1128/msphere.00173-23)
Supplement: TABLE S2 — The number of static clusters based on different single-nucleotide polymorphism cutoffs and the comparison between static clusters and dynamic groups. [file msphere.00173-23-s0006.docx]

Table S2. The number of static clusters based on different SNP cutoffs and the comparison between static clusters and dynamic groups.

| SNP cutoff | No. of static clusters | No. (%) of isolates assigned to a cluster | No. of the 59 dynamic groups matched a static cluster (one-to-one/several-to-one match)^a^ |
| --- | --- | --- | --- |
| 1 | 60 | 261 (35.8%) | 7 (4/3) |
| 2 | 72 | 335 (45.9%) | 11 (6/5) |
| 3 | 78 | 423 (57.9%) | 14 (6/8) |
| 4 | 78 | 444 (60.8%) | 20 (8/12) |
| 5 | 79 | 467 (64.0%) | 20 (9/11) |
| 6 | 73 | 486 (66.6%) | 26 (10/16) |
| 7 | 72 | 503 (68.9%) | 30 (13/17) |
| 8 | 68 | 518 (71.0%) | 32 (14/18) |
| 9 | 71 | 552 (75.6%) | 35 (13/22) |
| 10 | 65 | 568 (77.8%) | 39 (15/24) |
| 11 | 64 | 581 (79.6%) | 43 (19/24) |
| 12 | 58 | 590 (80.8%) | 46 (19/27) |
| 13 | 55 | 599 (82.1%) | 48 (20/28) |
| 14 | 49 | 607 (83.2%) | 52 (20/32) |
| 15 | 42 | 615 (84.2%) | 59 (16/43) |
| 16 | 41 | 622 (85.2%) | 59 (15/44) |
| 17 | 37 | 629 (86.2%) | 58 (10/48) |
| 18 | 37 | 634 (86.8%) | 57 (9/48) |
| 19 | 37 | 638 (87.4%) | 58 (10/48) |
| 20 | 36 | 647 (88.6%) | 56 (9/47) |
| 21 | 32 | 650 (89.0%) | 58 (8/50) |
| 22 | 29 | 653 (89.5%) | 59 (8/51) |
| 23 | 29 | 662 (90.7%) | 58 (7/51) |
| 24 | 27 | 666 (91.2%) | 59 (7/52) |
| 25 | 23 | 669 (91.6%) | 59 (7/52) |

^a^One-to-one match means that an individual static cluster contains a single dynamic group. Several-to-one match means that an individual static cluster contains two or more dynamic groups.
